# Supplementary material for: Spermine-Mediated Tolerance to Selenium Toxicity in Wheat (Triticum aestivum L.) Depends on Endogenous Nitric Oxide Synthesis
Source: Antioxidants (Basel). 2021 Nov 19;10(11):1835. doi: 10.3390/antiox10111835 (PMC8614684; doi:10.3390/antiox10111835)
Supplement: Supplementary file 1 [file antioxidants-10-01835-s001.zip › antioxidants-1466914-supplementary.pdf]

Table S1. The characteristics of the soil, and compost used in the experiment.

Properties	Value
**Soil**	
Texture analysis (%)	
Clay	48
Silt	28
Sand	24
Electrical conductivity [(1)], EC (mmho cm^{-1})	1.5
pH	7.6
Organic carbon (%)	0.9
$CaCO_3$	4.5
Soluble cations (meq L^{-1})	
Ca^{2+}	0.27
Mg^{2+}	0.26
Na^+	0.48
K^+	0.02
Soluble anions (meq L^{-1})	
Cl^-	0.5
SO_4^{2-}	0.13
Trace element (mg kg^{-1})	
Mn	4.7
Fe	3.2
Cu	1.3
Zn	1.2
Ni	0.7
Cd	0.1
**Compost**	
Organic carbon (%)	31.1
Organic matter (%)	53.1
Humic acid (%)	33.9
P-Olsen (ppm)	60
K_2O (ppm)	130

(1) Note: The data of electrical conductivity are expressed as mmho cm^{-1} considering that 1 dS/m = 1 mmho/cm.

Table S2. A list of primer used for gene expression analysis

**Primer Name**		**Sequence**	**Accession No.**
Metal tolerance protein (*MTP1*)	F	5′- ACGGCCATGACCATCACAATC - 3′	XM_021028261.1
	R	5′- GCTTGTCCTCTCCATGACCA - 3′	
Metal tolerance protein C3 (*MTPC3*)	F	5′- GGACAATCAGCTCCTCCAGA- 3′	XM_002878137.2
	R	5′-GAAGGTATATGCACGGACGG - 3′	
Phytochelatin synthase (*TaPCS1*)	F	5′- CAGACCACCATCCACGACTT - 3′	AF093752.1
	R	5′- ACAGCCTGTTCATTCCCTTT - 3′	
Heat shock protein (*HSP70)*	F	5′- CCCAGCGCCAGGCCACTAAGGAC-3′	AF005993
	R	5′- CAAAGCGAGCCCGTGTGATGGTA-3′	
Natural resistance associated macrophage protein (NRAMP1)	F	5′- GGATTCTCCTGGGTGCTGGGGTT- 3′	XM_015792143.2
	R	5′-GCAACAATCTACTCCCATGGGCC - 3′	
